# Targeting the Notch-Furin axis with 2-hydroxyoleic acid: a key mechanism in glioblastoma therapy

**DOI:** 10.1007/s13402-024-00995-x

**Published:** 2024-10-14

**Authors:** Raquel Rodríguez-Lorca, Ramón Román, Roberto Beteta-Göbel, Manuel Torres, Victoria Lladó, Pablo V. Escribá, Paula Fernández-García

**Affiliations:** 1 Department of Biology, Laboratory of Molecular Cell Biomedicine, University of the Balearic Islands, Palma de Mallorca, 07122 Spain; 2 R&D Department, Laminar Pharmaceuticals, C/Isaac Newton, Palma de Mallorca, 07121 Spain

**Keywords:** 2OHOA, Furin, Notch, Hes1, GBM, Melitherapy

## Abstract

**Purpose:**

Glioblastomas (GBMs) are highly treatment-resistant and aggressive brain tumors. 2OHOA, which is currently running a phase IIB/III clinical trial for newly diagnosed GBM patients, was developed in the context of melitherapy. This therapy focuses on the regulation of the membrane’s structure and organization with the consequent modulation of certain cell signals to revert the pathological state in several disorders. Notch signaling has been associated with tumorigenesis and cell survival, potentially driving the pathogenesis of GBM. The current study aims to determine whether 2OHOA modulates the Notch pathway as part of its antitumoral mechanism.

**Methods:**

2OHOA’s effect was evaluated on different components of the pathway by Western blot, Q-PCR, and confocal microscopy. Notch receptor processing was analyzed by subcellular fractionation and colocalization studies. Furin activity was evaluated under cleavage of its substrate by fluorescence assays and its binding affinity to 2OHOA was determined by surface plasmon resonance.

**Results:**

We found that 2OHOA inhibits Notch2 and Notch3 signaling by dual mechanism. Notch2 inhibition is unleashed by impairment of its processing through the inactivation of furin activity by physical association. Instead, Notch3 is transcriptionally downregulated leading to a lower activation of the pathway. Moreover, we also found that *HES1* overexpression highlighted the relevance of this pathway in the 2OHOA pharmacological efficacy.

**Conclusion:**

These findings report that the inhibition of Notch signaling by 2OHOA plays a role in its anti-tumoral activity, an effect that may be driven through direct inhibition of furin, characterizing a novel target of this bioactive lipid to treat GBM.

**Supplementary Information:**

The online version contains supplementary material available at 10.1007/s13402-024-00995-x.

## Introduction

Glioblastoma (GBM) is an aggressive primary brain tumor that is predominant in adults [1], comprising 70% of all gliomas [2], with an annual incidence of 0.59 to 5 per 100,000 population [3]. The lethality of GBM is in part due to its cellular and molecular heterogeneity, and its resistance to conventional therapies, with a median survival rate of 9 to 16 months associated with surgical resection, radiation and temozolomide (TMZ) chemotherapy [4]. Different molecular signaling pathways are involved in the uncontrolled cell proliferation and invasiveness of GBM. Among these, a disfunction of Notch signaling is known to suppress differentiation and the maintenance of stem cell properties [2], driving tumorigenicity and radioresistance [5]. This pathway is deregulated at multiple checkpoints in approximately three quarters of human GBMs [6] showing differential contribution of Notch receptors (1–4) to gliomagenesis with Notch2 playing a potential role [7–9]. Indeed, overexpression of *NOTCH2* has been related to poor prognosis and resistance to apoptosis death [10]. As well, *NOTCH3* overexpression has been reported in 71% of primary GBMs and it has been associated with an aberrant increase in the expression of its principal target gene, hairy and enhancer of split-1 (*HES1*) [6], which controls competence for astroglial and neuronal differentiation [11]. Moreover, several studies revealed a negative correlation between *HES1* expression and the survival of metastatic patients of different cancers [12, 13], producing drug resistance by maintaining cancer cells in a quiescent state [12]. Therefore, these major isoforms seem to play a predominant role in oncogenesis.

Notch receptors require to be processed by a furin-like protease to initiate the cascade of signaling. This enzyme, localized in the Golgi compartment, proteolytically cleaves the first S1 site of the Notch extracellular domain, completing its maturation and allowing it to be translocated to the cytoplasmic membrane [14]. Consequently, this proprotein convertase has been associated with pathophysiological processes like proliferation, migration and invasion in many types of cancer, such as astrocytoma [16], ovarian [16], colon [17] and lung [18] cancer. Indeed, this enzyme is responsible for generating the bioactive form of many substrates related to growth factor activity and their receptors, such as IGF1/2, insulin receptor (IR), c-MET, IGF1R [19, 20] or Notch [21, 22]. Therefore, it is not surprising that furin inhibition has been associated with the suppression of cell proliferation [15, 16, 23].

2OHOA (2-hydroxyoleic acid) is a synthetic monounsaturated fatty acid with anti-tumor efficacy that is currently being studied in a pivotal phase IIB/III clinical trial for newly diagnosed GBM patients (ClinicalTrials.gov identifier #NCT04250922). In xenograft models of human GBM, 2OHOA has stronger effect against the tumor and it better combatted relapses relative to TMZ, the current standard of care (SoC) for this disease [24]. Although its mechanism of action is still not fully elucidated, it is known that this bioactive lipid induces changes in the membrane lipid composition and organization [25, 26]. These alterations trigger modifications to several signaling pathways that influence proliferation, for example altering Ras translocation from the plasma membrane to the cytoplasm [24, 27, 28], as well as affecting pathways involved in differentiation or cell death and autophagy [24, 25, 27].

In the current study, we report that 2OHOA inhibits Notch2 and Notch3 signaling in GBM cell lines by impairing Notch2 processing through direct furin activity downregulation and repressing Notch3 transcription. These molecular events showed relevance in the 2OHOA mechanism of action as an antitumor drug highlighted by its main target, *HES1*. These findings may ultimately underlie the cell death induced by 2OHOA, suggesting that the inhibition of the Notch signaling pathway and furin proteases are relevant in the way 2OHOA acts in GBM.

## Materials and methods

### Cell culture and reagents

U-87 MG (ATCC^®^ HTB-14™; #70029548) and U-118 MG (ATCC^®^ HTB-15™; #61074619) cell lines were obtained from the American Type Culture Collection (Maassas, VA, USA; 19/01/2022 and 27/04/2016 respectively) while SF-295, SF-268, U-251, SNB-19, SNB-75 were purchased from Apointech SI (Salamanca, Spain). All experiments were performed with mycoplasma-free cells from Labclinics SA (Barcelona, Spain). All GBM cell lines were cultured under standard oxygen conditions at 37 °C in a humidified atmosphere of 5% CO_2_ and in RPMI 1640 medium (Sigma-Aldrich, St. Louis, MO, USA) supplemented with 10% Fetal Bovine Serum (FBS: Sigma-Aldrich), as recommended by the supplier. 2OHOA was obtained from Avanti (Alabaster, Alabama, USA) and reconstituted in ethanol: water (50:50). DAPT (N-[N-(3, 5-difluorophenacetyl)-l-alanyl]-s-phenylglycinet-butyl ester) was purchased from Santa Cruz Biotechnology (Heidelberg, Germany) and dissolved in DMSO.

### Cell transfection and gene expression silencing

The pCMV3-HES1 (HG17975-UT, Lot: G10JL08M019) was purchased from Sinobiological (Eschborn, Germany) and control vector was obtained by digesting the gene of interest inserted out the plasmid purchased. Transient transfection assays were carried out with Lipofectamine™ 2000 (Thermo Fisher Scientific, Inc., Waltham, MA, USA) according to the manufacturers’ instructions. Characterization of Notch homolog proteins (1–3) was performed by specific small interfering RNA (siRNA) assays. Cells were transfected with 20 nM of commercial Notch1, Notch2 (Santa Cruz Biotechnology, Heidelberg, Germany) or Notch3 siRNA (Thermo Fisher Scientific, Inc., Waltham, MA, USA) for 72 h using Lipofectamine™ RNAiMAX protocol (Thermo Fisher Scientific, Inc., Waltham, MA, USA). The non-specific siRNA (Thermo Fisher Scientific, Inc., Waltham, MA, USA) was used as control.

### Cell viability assay (trypan blue exclusion)

Cell viability was assessed by Trypan blue staining [29]. U-118 MG and U-87 MG glioblastoma cells were plated in 6-well plates at densities of 1.5 × 10^4^ cells/cm^2^ for transient transfections. After overnight incubation, the cells were transfected with a cocktail of 2 µg of plasmid, 2 µl Lipofectamine 2000 and 150 µl of Opti-MEM (Thermo-Scientific), maintained separately at room temperature for 10 min and then mixed for 20 min to achieve complex formation. The transfection cocktail was then added drop-wise to the cultured cells and 24 h post-transfection, the culture medium was replaced and the cells were exposed to 2OHOA (200 µM) or the vehicle alone (50% ethanol) for 48 h. After this treatment, trypan blue staining was performed by mixing the cell suspension with trypan blue (Invitrogen) in a 1:1 ratio and pipetting the cells into a BRAND^®^ counting chamber (Sigma).

### Real‑time quantitative reverse transcription PCR (qRT-PCR)

Total RNA from glioma cells used in the cell viability and siRNA assays was extracted using the TriPure Isolation Reagent (a monophasic solution of phenol and guanidine thiocyanate at pH 4: Roche), according to the manufacturer’s instructions, and it was quantified using a Nanodrop2000 Spectophotometer at 260 nm (Thermo Scientific). The High-Capacity cDNA Reverse Transcription Kit (Applied Biosystems, Barcelona, Spain) was used to synthesize cDNA from the total RNA (1 µg) recovered. The target cDNA was amplified from each sample (in triplicate) by quantitative Real-Time PCR (qRT-PCR) in 10 µL reactions using a Step One Plus Real Time PCR thermal cycler (Applied Biosystems). The reaction mixture contained 1× of SYBR^®^ Premix Ex Taq™ II (Tli RNaseH Plus, Clontech, Saint-Germain-en-Laye, France), 200 ng µl of cDNA, 0.2 µM of each forward (Fw) and reverse (Rv) primer, and 1× ROX Reference Dye (50×). The primer sequences used in the PCR assays are described in Table 1 and RPL13 was used to normalize the quantifications to the amount of mRNA in each sample. A standard curve of known cDNA concentrations was used to calculate the basal mRNA concentrations. Serial dilutions were used in two replicates to establish the standard curve, starting from 50 ng of cDNA and measured using the Step One Plus Real Time PCR System (Applied Biosystem). Based on the standard curve the relative amount of mRNA was determined using StepOne Software v2.3.

**Table 1:** Sequence of primers used in the PCR assays

Primer	Sequence 5′ → 3′
JAGGED1 Fw	ATGCGTTCCCCACGGAC
JAGGED1 Rv	GTTCTGCAGCTCCCCGTT
NOTCH1 Fw	TGAATGGCGGGAAGTGTGAA
NOTCH1 Rv	ATAGTCTGCCACGCCTCTG
NOTCH2 Fw	TACAGTTGTCGCTGCTTGCC
NOTCH2 Rv	CGACGAAGGTTTCACAGTGC
NOTCH3 Fw	CAGTGTGAACTCCTCTCCCC
NOTCH3 Rv	GGTGCAGATACCATGAGGGC
HES1 Fw	AGGCGGACATTCTGGAAATG
HES1 Rv	TCGTTCATGCACTCGCTGA
CD3 Fw	ACCAGCATTCCTTTTGAACG
CD3 Rv	TTTTAGAGGGACCCCAATCC
RPL13 Fw	CCCCCAAGAAGGGAGACAGT
RPL13 Rv	GGAGACTAGCGAAGGCTTTGA

### Protein sample preparation and Western blot analysis

Glioma cell lines were seeded in 6 cm^2^ plates with RPMI 1640 medium supplemented with 10% FBS at densities of 1.5 × 10^4^ cells/cm^2^ for transfection, or in P60 dishes at 3.75 × 10^4^ cells/cm2 for the time course experiments. Transfection assays were processed as described previously. For time course experiments, cells were treated with 2OHOA (200 µM) for 6, 12, 24–48 h. The cells were then rinsed twice with phosphate-buffered saline (PBS, pH 7.6) and the proteins isolated in lysis buffer (300 µL): 10 mM Tris-HCl supplemented with 2 mM EDTA, 1% SDS, 5 mM Protease Inhibitor Complete (ROCHE) and 1 mM Sodium Orthovanadate. Cell suspensions were ultrasonicated for 3 cycles of 20 s at 50 W in a Branson Sonifier^®^ W-450D (USA) and the protein recovered was quantified with a Lowry protein assay [30], RC DC™ (BioRad). The samples (30 µg) were loaded onto acrylamide gels and immunoblotted as reported in [31] with minor variations. The nitrocellulose membranes were probed overnight at 4°C with the primary antibodies against Notch2 Intracellular domain (Hybridoma Bank, Iowa City, Iowa), Notch3 (Santa Cruz Biotechnology, Heidelberg, Germany) and Hes1 (Santa Cruz Biotechnology, Heidelberg, Germany) diluted 1:1,000. After washing, the membranes were exposed for 1 h at room temperature to a goat anti-mouse IgG secondary antibody diluted 1:10,000 (Li-cor, Biosciences, Lincoln, NE, USA). Antibody binding was detected using near infrared scanning at 700 nm (Odissey, Li-cor Biosciences, Lincoln, NE, USA), and the specific signals were quantified by integrated photodensitometry and normalized to the α-Tubulin signal (1:10,000, Sigma) used as a reference protein.

### Immunofluorescence analysis

GBM cell lines were seeded onto 25 mm round coverslips in 24-well plates containing 1 mL of RPMI 1640 medium supplemented with 10% FBS at a density of 1.5 × 10^4^ cells/cm^2^ for U-87 MG GBM cells. After overnight cell attachment, the cells were treated with 2OHOA (400 µM) for 48 h and subsequently fixed at room temperature for 15 min with 4% paraformaldehyde (PFA) in phosphate buffer (PB). After washing twice with PBS, 0.1% Triton X-100 (Sigma) was added to permeabilize the fixed cells for 10 min at room temperature. The cells were then incubated with ammonium chloride (100 mM: Sigma) at room temperature for 10 min and they were then stained overnight with primary antibodies against Notch3 (Santa Cruz Biotechnology, Heidelberg, Germany), Notch2 (BioLegend, Barcelona, Spain), Hes1 (Santa Cruz Biotechnology, Heidelberg, Germany) and Giantin (Biolegend, Barcelona, Spain), all diluted 1:100. Subsequently, the coverslips were rinsed 10 times in 0,1% Tween-Tris-buffered saline (T-TBS) and incubated at room temperature for 1 h with the appropriate Alexa Fluor^®^ 488 or Alexa Fluor^®^ 598 conjugated IgGs (Invitrogen, Carlsbad, CA, USA). The cells were then washed again 10 times in T-TBS and stained with DAPI (Sigma, Darmstadt, Germany) for 5 min at room temperature. After rinsing 10 times with PBS, the coverslips were mounted in Vectashield (Hardse: VECTOR) and visualized at 40x or 63x under a confocal fluorescence microscope (Leica Microsystem TCS SPE). The data were analyzed using Fiji-ImageJ software and for co-localization, Pearson and Manders’ coefficients were obtained from eight representative images from at least two independent experiments using the JACoP (Just another co-localization plugin) co-localization tool [32].

### Subcellular fractionation assay

Fractionation was carried out on ice and using differential centrifugation at 4 °C. Cells were collected in PBS and recovered by centrifugation at 260 rcf for 3 min at 4 °C. The supernatant was replaced with 10 mM Tris (pH 7.6) buffer containing 1 mM EDTA, 1 mM EGTA and 5 mM of the Complete Protease Inhibitor mix. After a 10 min incubation, the cell suspension was homogenized through a syringe 20 times and the solution was centrifuged to pellet the nuclei (730 rcf, 10 min). After centrifuging the supernatant to pellet any debris (20,000 rcf, 3 min), the cytoplasmic fraction was obtained by centrifugation at 20,000 rcf for 30 min. The pellet was washed with hypotonic buffer and centrifuged (20,000 rcf, 30 min), and after resuspending the pellet in 150 mM citrate (pH 6.4) and 0.1 M alkaline (pH 12.6) buffers for 10 min on ice, and for 30 min in a mini labroller rotator (Labnet H550, USA), the material was centrifuged at 20,000 rcf for 60 min. Finally, the supernatant (soluble membrane fraction) was neutralized with 1 M Tris HCl buffer (pH 6.8), and the pellet (membrane fraction) was resuspended in 1 M Tris HCl (pH 7.4) containing 5% SDS and passed through a syringe 10 times. Each fraction sample was analyzed in immunoblots probed with a 1:1,000 dilution of an anti-Na^+^/K^+^-ATPase α (Santa Cruz Biotechnology, Heidelberg, Germany) antibody as a membrane marker or an anti-Lamin B1 antibody (Santa Cruz Biotechnology, Heidelberg, Germany) as a nuclear marker, and with an anti-α-Tubulin (1:10,000) antibody as a cytoplasmic marker.

### Xenograft tumor model

All the experimental procedures involving animals were carried out in accordance with the animal welfare guidelines of the European Union (86/609/EEC) and with the authorization of the Institutional Committee for Animal Research at the University of the Balearic Islands (RD 53/2013). U-118 MG cells were incubated in RPMI 1640 medium supplemented with 10% FBS and 8-10-week-old immunodeficient NUDE Swiss Crl: NU (Ico)-Foxn1nu mice (Charles River Laboratories, France) were housed in a specific-pathogen-free facility. To generate the xenograft glioma models, 7.5 × 10^6^ U-118 MG cells were subcutaneously inoculated into the animal’s dorsal area and 10 days after inoculation, the animals were randomly divided into groups that received 2OHOA (200 mg/Kg daily, p.o.) suspended in 0.5% (w/v) in carboxymethylcellulose (CMC, Sigma, MO) or the vehicle alone (CMC), administered using a graduated syringe with a metal cannula over 42 days. Tumor volumes were calculated using the equation:

$${\mathrm{Volume~}}\left[ {{\mathrm{m}}{{\mathrm{m}}^3}} \right]=\frac{{{W^2}\times{\mathrm{L}}}}{2}$$

where W is the tumor width and L its length. At the end of the treatment, the animals were sacrificed by decapitation and 8–15 mg of the central part of the tumors was homogenized with Tripure^®^ in a grinder and using ultrasound. RNA was isolated as described above.

### Surface plasmon resonance

The binding affinities of furin to OA and 2OHOA were investigated by surface plasmon resonance (SPR) carried out at 25 °C on a Biacore X100 apparatus (GE Healthcare, Chicago, IL, USA). For immobilization, the standard Cytiva amine coupling kit was used on a CM5 sensor chip, with HBS-EP as running buffer (10 mM HEPES at pH 7.5, 150 mM NaCl, 3 mM EDTA, and 0.005% Tween 20) following the manufacturer’s instructions. Subsequently, 28 µg/mL of commercial recombinant Human Furin (450 − 47, Preprotech) diluted in 10 mM of sodium acetate at pH 4.5 was injected until the immobilization level reached approximately 1000 response units (RUs). Unreacted activated carboxyl groups on the sensor chip surface were deactivated for 400 s with 1 M ethanolamine (pH 8.5). The other reference flow cell left in the chip was treated in the same way but without protein, and the response of the control was subtracted from each sample dataset. 2OHOA and OA were dissolved in running buffer (PBS-EP+) at concentrations from 50 to 1.8 µM and injected over the chip at a flow of 20 µL/min during a 45 s association period, followed by a 70 s dissociation. The sensor chip surface was regenerated with two additional washes with running buffer after testing.

Affinity calculations were performed at the concentrations indicated, 4 s after stopping the injection, using points at the steady state of the interaction. Concentrations higher than 50 µM lead to a response level higher than the theoretical Rmax (5 RU) calculated for a 1:1 model interaction. This response was probably due to analyte aggregation as these concentrations are above the critical micelle concentration of the molecules (30 µM).

### Furin activity assay

Furin activity was evaluated in vitro using a fluorimetric “Sensolyte Rh110 Furin activity assay” (ANASPEC), following the manufacturer’s instructions. The effect of 2OHOA on Furin activity was evaluated at 200 and 400 µM 2OHOA, prepared in the vehicle (ethanol: water, 50:50). A peptidyl chloromethylketone (Decanoyl-Arg-Val-Lys-Arg-CMK) was used at 0.2 µM as a positive control of furin inhibition. Test compounds (1:10 per mix solution) were pipetted into a black 96-well plate with a non-binding surface. The fluorescence signal was measured continuously at Ex/Em = 490/520 nm every 5 min over 60 min on a Fluostar Omega microplate reader (BMG Labtech, Germany). The rhodamine 110 (Rh110) fluorescence signal was subtracted from the readings of the remaining samples to obtain the relative fluorescence unit (RFU), which were analyzed against time for each sample, taking the RFU of Recombinant human furin mixed with its substrate as 100%.

Furin like protease activity was measured in vivo using Boc-Arg-Val-Arg-Arg-AMC (Thermo Fisher Scientific, Inc., Waltham, MA, USA) as a substrate for FLP enzymes. The cells were seeded in 96-well plates for 24 h (15 × 10^3^ cells) and treated with increasing concentrations of 2OHOA (100, 200, 300, 400, 600 and 800 µM) for 48 h, or with 2OHOA (400 µM) for 1 and 3 h to determine the short-term effects. The growth medium was then replaced by 50 µl RPMI 1640 medium without phenol red and containing 0.25% Triton X-100 to permeabilize the cells. Fluorescence was measured at Ex/Em = 389/460 nm 1 h after the addition of the substrate (100 µM). The aforementioned furin inhibitor (100 nM) was used as a positive control of the inhibition of protease activity. A crystal violet assay was performed in parallel to normalize the data to cell density and the RFU were analyzed against the concentration of the test compounds.

### Statistical analysis

The data are shown as the mean ± SEM from at least 2 independent experiments with duplicate or triplicate samples for in vitro assays and the number of animals is indicated for the in vivo assays. A two-sided Student’s *t*-test with Welch’s correction or a non-linear regression were used to compare the experimental groups. The differences between groups were analyzed as the % relative to the untreated controls and statistical significance was considered at *p* < 0.05: *p < 0.05, **p < 0.01 and ***p < 0.001. Delta Cts were calculated using the formula:

$$\Delta {\mathrm{Ct~}}={\mathrm{~Ct~}}\left( {{\mathrm{gene~of~interest}}} \right){\mathrm{~}}-{\mathrm{~Ct~}}\left( {{\mathrm{housekeeping~gene}}} \right)$$

The correlations between the IC_50_ values and mRNA levels were analyzed using a Pearson’s correlation test.

## Results

### *HES1* expression is involved in the antitumor effect of 2OHOA on glioblastoma

**Fig. 1:**
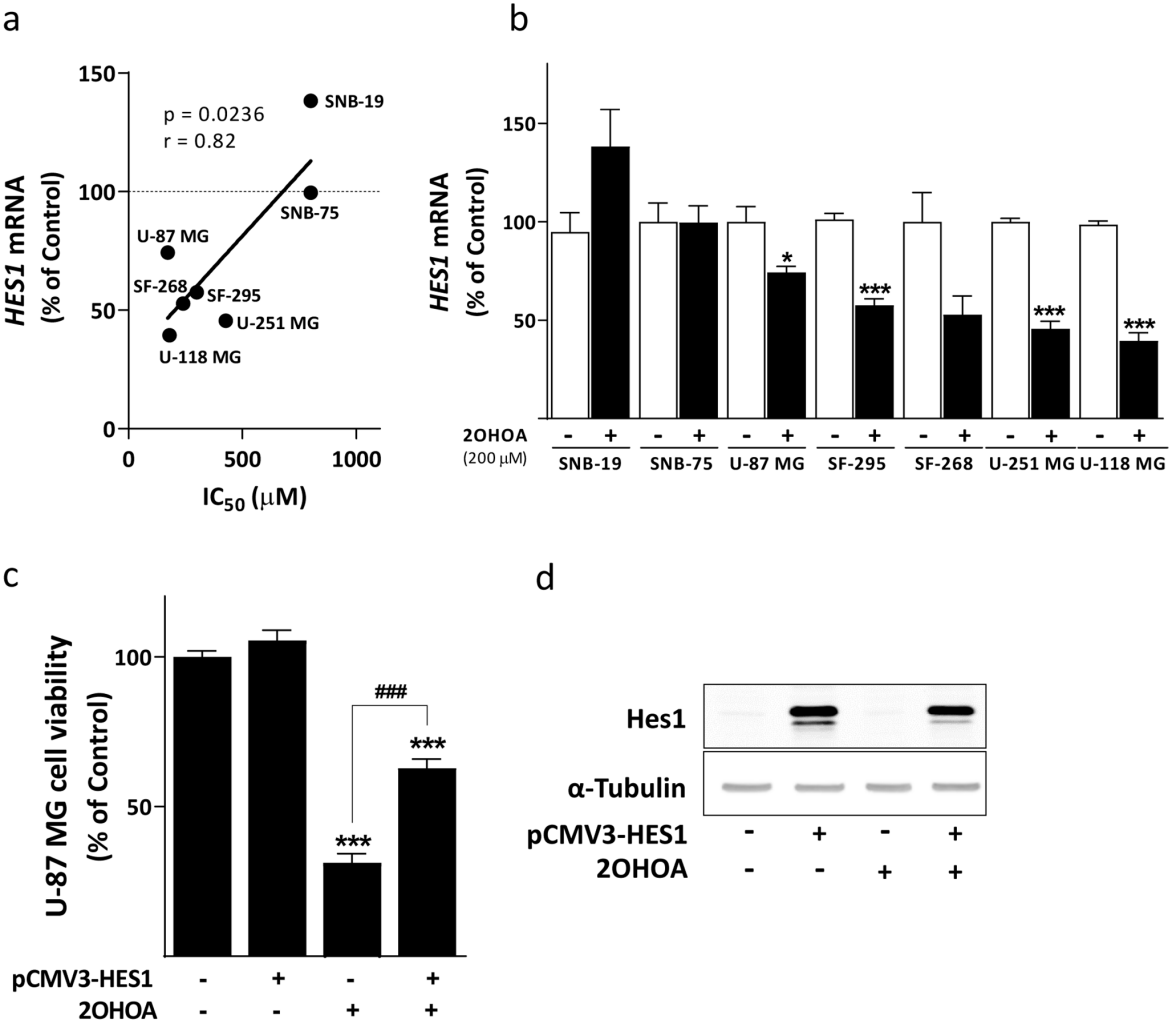
*HES1* expression is involved in the mechanism of action of 2OHOA. **a** Positive correlation between the IC_50_ and *HES1* expression in response to a 24 h exposure to 2OHOA (200 µM) expressed as a % of the control ± S.E.M (*p* = 0.00236). The values obtained by qRT-PCR from at least two independent experiments with four replicates each. **b***HES1* mRNA levels in the presence of 2OHOA (200 µM, 24 h) or the vehicle alone analyzed through multiple *t* tests: *p < 0.05, **p < 0.01, ***p < 0.001. The results from at least three independent experiments, with three replicates each, expressed as the % of control ± S.E.M. **c** Viability of U-87 MG cells transfected with 1 µg of empty plasmid (control) or pCMV3-HES1 for 24 h prior to 2OHOA treatment (200 µM for 48 h) as determined by trypan blue exclusion. The data are expressed as the % of the control ± S.E.M of three independent experiments with three replicates each. The statistical analysis was performed with a *t*-test with Welch’s correction relative to cells transfected with the empty plasmid: **p < 0.01, ***p < 0.001 or pCMV3-HES1: ^###^*p* < 0.001. **d** Transfection efficiency of pCMV3-HES1 into U87-MG cells as seen in Western blots

Due to the relevance of *HES1* in the proliferation and survival of GBM cells [33], we investigated its possible involvement in the effects of 2OHOA on these tumors. We assessed whether the response of different glioma cell lines to 2OHOA might be related to their basal *HES1* expression. A comparison between basal *HES1* mRNA expression and the half maximal inhibitory concentration (IC_50_) values of 2OHOA failed to detect such a correlation (Table S1a). However, a significant correlation was shown between the IC_50_ values and the inhibition of *HES1* mRNA expression when cell lines were exposed to 2OHOA (200 µM) for 24 h (*p* = 0.0236, *r* = 0.82: Fig. 1a, Table S1a), resulting in lower *HES1* expression relative to the untreated cells (U-87 MG, 25.68% ± 3.01; SF-295, 43.59% ± 3.31; SF-268, 47.23% ± 9.59; U-251 MG, 54.45% ± 3.9; U-118 MG, 59.12% ± 4.19). By contrast, there was no reduction in *HES1* mRNA levels in cells that did not respond to 2OHOA (IC_50_ ≥ 600 µM: SNB-19, 138.30% ± 18.85; SNB-75, 99.59% ± 8.51), suggesting the relevance of this gene in the effects of 2OHOA on GBM (Fig. 1b, Table S1b).

Considering the significant positive correlation between the shift in *HES1* mRNA expression in response to treatment and the cell’s pharmacological sensitivity based on their IC_50_ values, the relevance of Hes1 levels to the effect of 2OHOA on cell viability was evaluated. Transient transfection of pCMV3-HES1 was performed in presence or absence of 2OHOA. Whereas the bioactive lipid decreased the viability in both cell lines (U-87 MG 69.76% ± 5.19; U-118 MG 56.30% ± 6.05), *HES1* overexpression showed an increase in its viabilities, 17.10% ± 7.66 for U-118 MG cells but non-statistically significant in U-87 MG cells relative to the untreated cells (Fig. 1c, Fig. S1a). This difference could be related to the lower Notch3 basal activation of this pathway in U-118 MG cells relative to the U-87 MG cells (Fig. S2a). Interestingly, *HES1* overexpression partially inhibited the effect of 2OHOA on cell viability in U-87 MG (31.55% ± 4.35) and U-118 MG (40.02% ± 5.66) cells and hence, *HES1* appeared to be involved in the effects of 2OHOA on these cells. The transfection efficiencies in these studies were confirmed by analyzing the Hes1 protein in immunoblots (Fig. 1d, Fig. S1b).

### 2OHOA efficacy is correlated with *HES1* expression in vivo

**Fig. 2:**
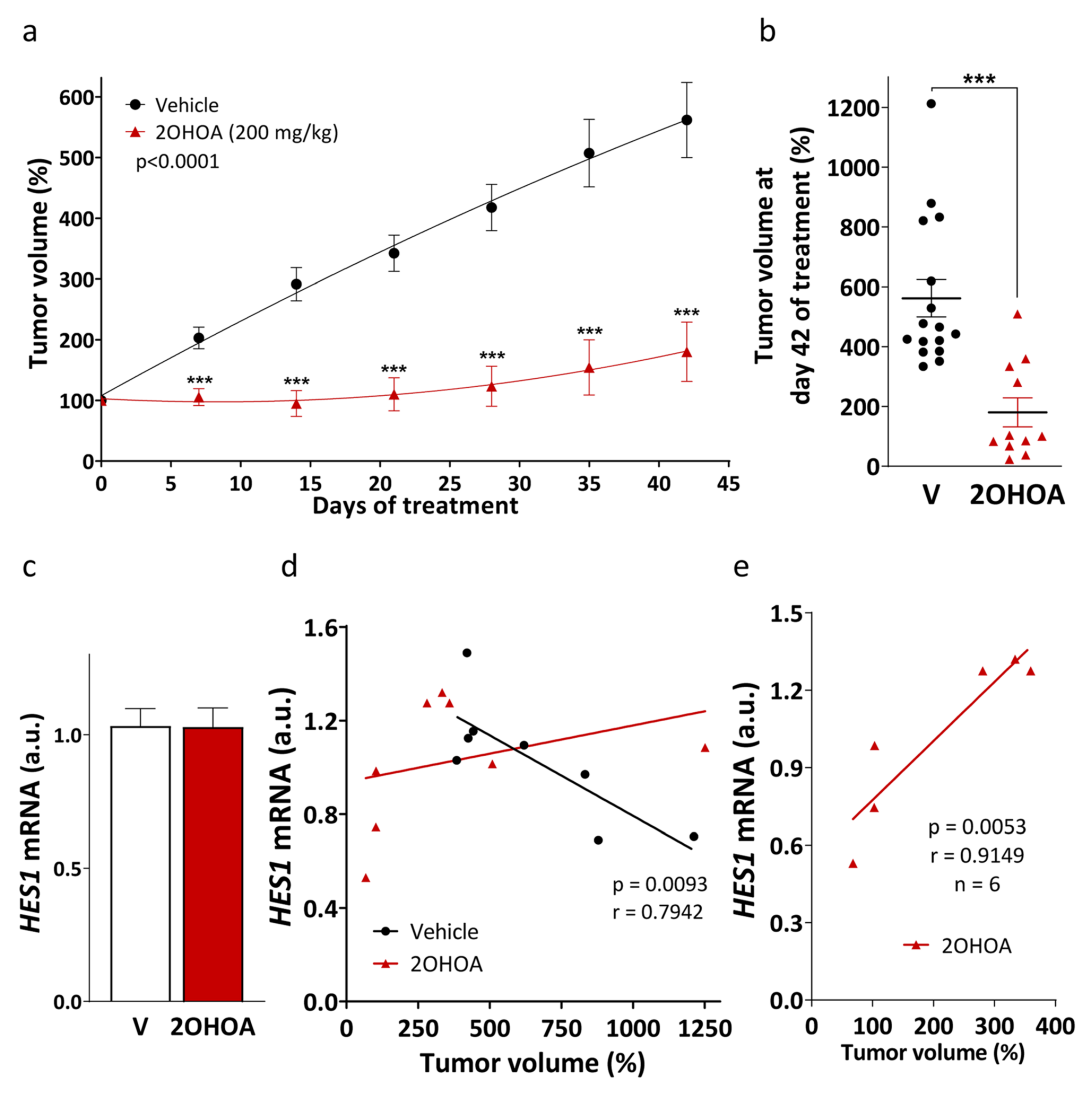
The tumor volume in response to 2OHOA is correlated with *HES1* expression. **a** The effect of 2OHOA (200 mg/kg, p.o., daily) and vehicle (V) administration on U-118 MG-derived xenograft tumor growth in mice over 42 days (control, 16 tumors; 2OHOA, 11 tumors). The data are expressed as the mean ± S.E.M and analyzed by multiple *t* tests: ***p < 0.001. **b** Tumor volumes after a 42-day treatment (mean ± S.E.M). **c***HES1* expression in U-118 MG cell-derived tumors determined by qRT-PCR after a 42-day exposure to 2OHOA or the vehicle alone. The data are presented as the mean ± SEM of 8 tumors/group analyzed in duplicate. **d** Representation of the *HES1* expression relative to the volume of the tumors from vehicle or 2OHOA-treated mice. The linear regression slope is opposite in the mice that received 2OHOA compared to those administered the vehicle alone. The correlation between *HES1* expression and tumor volume is significant in the vehicle samples (*p* = 0.0093 and *r*=−0.7942). **e** Representation of *HES1* expression with respect to tumor volume in those tumors that responded to 2OHOA. The correlation between *HES1* expression and tumor volume is significant as an indicator of 2OHOA efficacy and tumor progression (*p* = 0.0053 and *r* = 0.9149)

The efficacy of 2OHOA was confirmed using xenograft tumors generated from U-118 MG cells. Tumor volume was measured weekly and from day 7 onwards the tumors grew more slowly (105.6% ± 14.02) in mice that received 2OHOA (200 mg/kg, p.o. daily) than in those that did not (203.19% ± 18.48: Fig. 2a). After 42 days of 2OHOA treatment, the tumor volume was reduced by 67,93% compared to mice that received the vehicle alone (Fig. 2b). At the end of the experiment, the xenograft tumors were excised and *HES1* RNA expression was evaluated by qRT-PCR as a marker of proliferation. *HES1* expression did not differ significantly in tumors from mice that received 2OHOA compared to those that received the vehicle alone (Fig. 2c). Interestingly, while a strong negative correlation was showed between *HES1* expression and tumor volume in mice that received the vehicle alone (*p* = 0.0093 and *r*=-0.7942), a positive correlation in the mice that received 2OHOA was revealed (Fig. 2d, Table S2). Indeed, a significant positive correlation between *HES1* expression and tumor volume (*p* = 0.0053 and *r* = 0.9149) was evident in 2OHOA-sensitive tumors, considering the smaller 2OHOA-treated tumors than the smallest tumors in the mice that received the vehicle alone (Fig. 2e). These results appear to reflect an importance of *HES1* expression in the response of GBM xenografts to 2OHOA in vivo in immunosuppressed mice.

### 2OHOA inhibits Notch signaling

In the light of these results and taking into consideration that *HES1* is a classic target of Notch signaling, the effect of 2OHOA on the activation of this pathway was assessed through the levels of its effector, NICD. Characterization of Notch homolog proteins were carried out by small interfering RNA (siRNA) transfection assays. Revealing against specific antibodies of Notch2 and Notch3, we identified the resulting fragments of its processing: the Notch2 receptor or full-length (FL ≈ 300 KDa), Notch2 transmembrane domain (TM ≈ 120 KDa), Notch 2 intracellular domain (NICD2 ≈ 90 KDa) as well as Notch3 FL (≈ 275 KDa), Notch3 TM (≈ 90 KDa) and NICD3 (≈ 80 KDa) in their respective siNotch-mediated knockdown cells (Figure S2B, C). Notch1 mRNA was silencing due to its highest homology with Notch2 showing no detection of Notch1 when revealing with anti-Notch2 antibody. Efficiency of the siRNAs-mediated knockdown was confirmed by measuring mRNA expression levels of *NOTCH1*, *NOTCH2* and *NOTCH3* (Fig. S2d). Although Notch2 pathway is the most activated homologous protein in these GBM cells (Fig. S2a), relevance of inhibiting both pathways (Notch2 and Notch3) to downregulate Hes1 protein was also determined when targeting Notch2 or Notch3 relative to non-specific siRNA transfected cells (Fig. S2e).

**Fig. 3:**
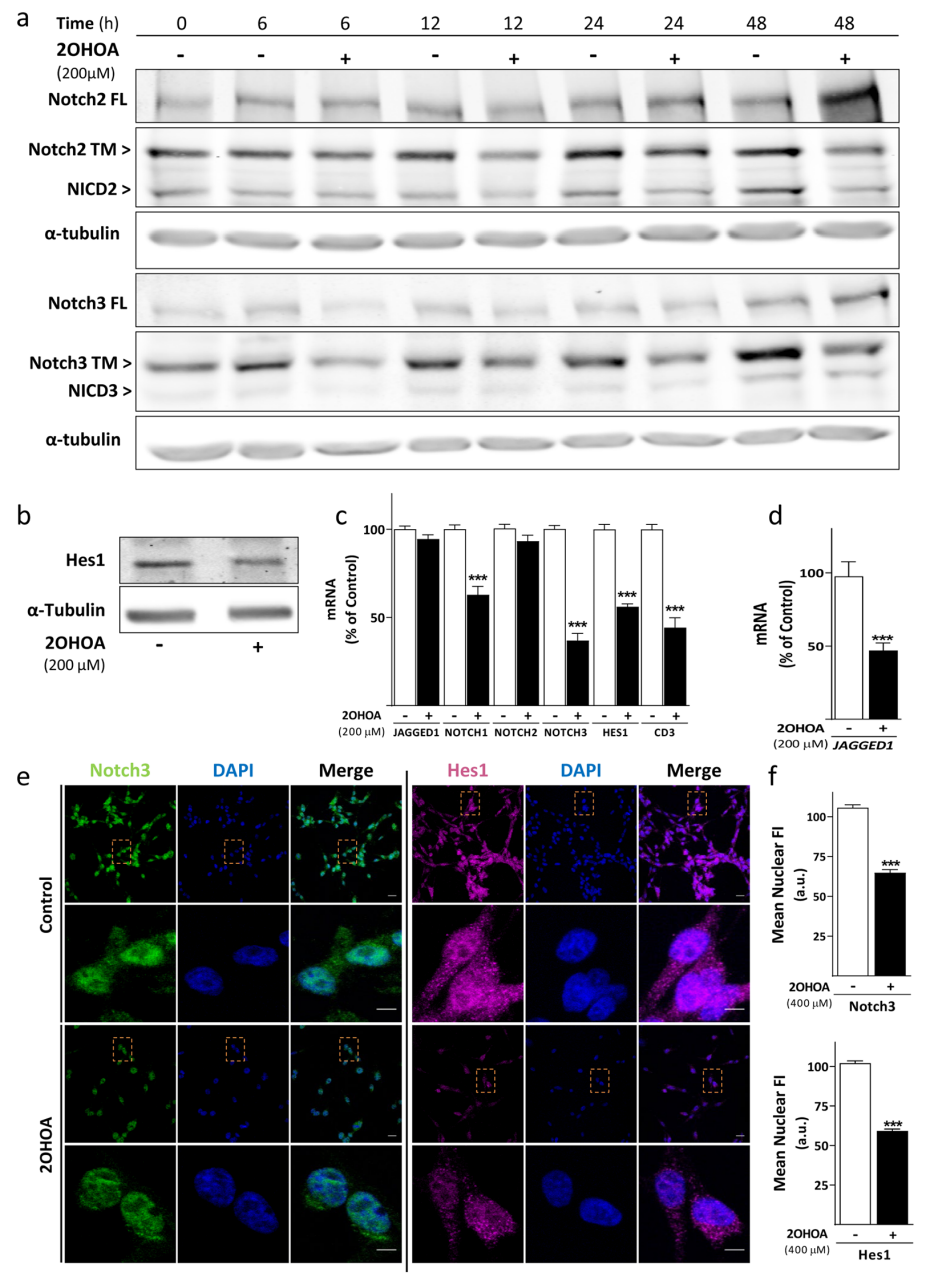
2OHOA inhibits Notch signaling pathway. **a** Representative immunoblots of Notch2/3 FL (full length), Notch2/3 TM (transmembrane domain) and NICD2/3 (Notch intracellular domain) proteins after exposure of U-87 MG cells to 2OHOA (200 µM) for 6, 12, 24 and 48 h (four independent experiments with two replicates each). **b** The Hes1 protein levels after exposure to 2OHOA (200 µM) for 48 h as an indicator of its pharmacological effect in U-87 MG cells. **c** The mRNA expression of *JAGGED1*, *NOTCH1*, *NOTCH2*, *NOTCH3*, *HES1* and *CD3* genes in U-87 MG treated for 48 h with 2OHOA (200 µM) or the vehicle alone. The values are presented as the mean ± SEM of at least four independent experiments analyzed in triplicate. **d***JAGGED1* mRNA levels in U-87 MG cells exposed to 2OHOA (200 µM) or the vehicle alone for 72 h. The results are from three independent experiments with three replicates each and they are expressed as the % of the control ± SEM. **e** Cell localization of Notch3 (left panels) and Hes1 (right panels) in U-87 MG control cells (row 1, 2) or those exposed to 2OHOA (400 µM) for 48 h (row 3, 4). Images were taken at 40× magnification. Scale bar: 20 μm (row 1, 3), 5 μm (row 2,4). **f** Nuclear fluorescence intensity of Notch3 (upper graph) or Hes1 (lower graph) in two independent experiments. The statistical analysis was performed with a *t*-test with Welch’s correction relative to the untreated cells: ***p < 0.001

Once homolog members of the Notch family were determined, a time course of NICD was performed to evaluate its inhibition in U-87 MG and U-118 MG cells exposed to 200 µM of 2OHOA for 6, 12, 24 and 48 h. A decrease in Notch3 FL, Notch3 TM and NICD3 was obtained after a 6 h exposure to 2OHOA (first time tested) in U-87 MG cells. However, the effect of 2OHOA was later pronounced in U-118 MG probably due to the fact that it presents half activation of this isoform in comparison to U-87 MG. According to Notch2, an accumulation of the receptor was shown after 48 h exposure to 2OHOA with the ensuing decrease of Notch2 TM in both cell lines and NICD2 in U-87 MG (Fig. 3a, Fig. S3a). Hence, 2OHOA can inhibit Notch2 and Notch3 signaling in both these cell lines. Effect of 2OHOA was confirmed by a decrease in Hes1 protein in U-87 MG cells (50.28% ± 9.8: Fig. 3b) and in U-118 MG cells (40.9% ± 7.5: Fig. S3b).

The expression of genes related to Notch signaling pathway was also evaluated by qRT-PCR in U-87 MG and U-118 MG cells treated for 48 h with 2OHOA (200 µM). The expression of these genes was reduced significantly relative to the respective untreated cells in both cases: U-87 MG cells—*NOTCH1* 37.09% ± 5.3, *NOTCH3* 62.79% ± 4.52, *HES1* 43.87% ± 3.2 and *CD3* 55.69% ± 6.18 (Fig. 3c); and U-118 MG cells—*JAGGED1* 59.21% ± 4.92, *NOTCH1* 27.07% ± 4.96, *NOTCH2* 17.66% ± 5.73, *NOTCH3* 79.18% ± 2.49, *HES1* 67.37% ± 3.81 and *CD3* 65.51% ± 3.3 (Fig. S3c). While in U-87 MG cells the expression of the *JAGGED1* ligand was not significantly lower at 48 h, it was at 72 h (50.72% ± 11.26: Fig. 3d). Interestingly, the expression levels of *NOTCH2* were not reduced in presence of 2OHOA in U-87 MG cells suggesting that 2OHOA inhibits Notch2 and Notch3 signaling pathway by dual mechanism. Overall, the genetic and protein analysis indicate that 2OHOA negatively regulates Notch signaling by reducing the amount of Notch3 protein and preventing Notch2 processing but also, by repressing the transcription of the genes encoding its ligand and receptors. Consequently, it is likely that other related signaling pathways are also inhibited and as a result, so are their target genes, producing almost complete inhibition of the pathway.

To corroborate whether 2OHOA decreased the amount of the effector NICD3 in the nucleus as seen in immunoblot, U-87 MG cells were exposed to 2OHOA (400 µM) for 48 h and the ensuing immunofluorescence assays revealed a significant reduction of NICD3 in the nucleus of 2OHOA-treated cells (38.65% ± 2.57) relative to the controls (Fig. 3e, f). Accordingly, there was also a depletion of nuclear Hes1 (41.99% ± 1.96) following exposure of U-87 MG cells to 2OHOA (Fig. 3f). Indeed, the effect of 2OHOA on cell viability (Fig. 1c) was similar to the decrease of these nuclear proteins, supporting the relevance of Hes1 and Notch signaling to the pharmacological efficacy of 2OHOA.

### 2OHOA prevents Notch2 processing by furin-like enzyme inhibition

**Fig. 4:**
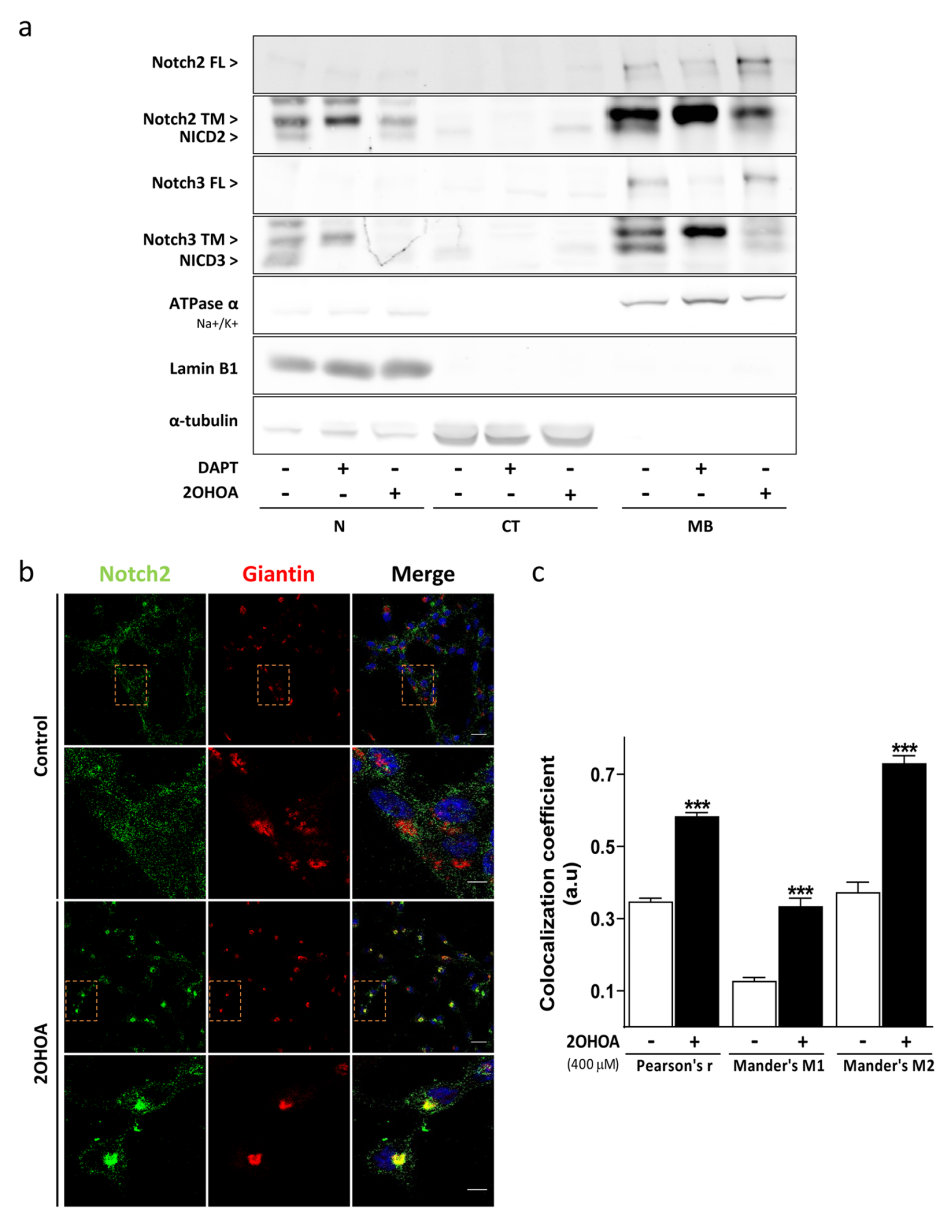
2OHOA prevents Notch2 processing and trafficking. **a** Representative immunoblot of the U-87 MG subcellular fractionation from six independent experiments, with two replicates each. The distribution of the Notch protein: Notch2/3 FL (full length), Notch2 TM (transmembrane domain) and NICD3 (Notch intracellular domain) in cells maintained for 48 h in the presence or absence of 2OHOA (400 µM) or DAPT (10 µM, 24 h), a positive control for the loss of NICD, is shown in the different cellular compartments: N, nucleus; CT, cytosol; and MB, total membranes. The efficiency of fractionation was assessed by analyzing reference proteins from the different compartments: Laminin B1 for the nucleus, tubulin for the cytosol, and ATPase α (Na^+^/K^+^) for the membranes. **b** U-87 MG immunofluorescence staining and co-localization analysis (**c**) of Notch2 (green channel) with Giantin (Golgi marker, red channel) in response to 400 µM for 48 h of vehicle (row 1, 2) or 2OHOA (row 3, 4). Images were taken at 63× magnification. Scale bar: 20 μm (row 1, 3), 5 μm (row 2, 4). Pearson correlation coefficient indicate the probability that the pixels of both channels coincide. Manders’ coefficients of M1 (overlap of Notch2 with Giantin) and M2 (overlap of Giantin with Notch2 signal). The values are given as the mean ± SEM and significant differences between the treatment and vehicle were analyzed with a Welch’s *t*-test: ***p < 0.001

In view of the accumulation of the Notch2 receptor triggered by 2OHOA (Fig. 3a, Fig. S3a), its distribution in the different cellular compartments of both GBM cell lines was evaluated by subcellular fractionation, separating the nucleus, cytosol and membranes. Cells were treated with 2OHOA (400 µM, 48 h), the vehicle alone or with DAPT (100 µM, 24 h), a positive control for the absence of the NICD fragment. A striking accumulation of Notch2 receptor in the membrane fraction of both U-87 MG and U-118 MG cells was shown, with the ensuing decrease of NICD2 in the nuclear fraction, suggesting that 2OHOA prevents receptor processing in the Golgi, ER or plasma membrane. The aggregation of Notch2 full-length along with its transmembrane domain, were also induced by 2OHOA in the nuclear fraction of U-118 MG cells due to the contamination of this compartment by the membrane fraction, as confirmed by the presence of ATPase as a membrane marker. The NICD3 fragment was not detected in the nuclear fraction of either cell line following exposure to 2OHOA and relative to the control cells, hinting that 2OHOA inhibits Notch signaling by dual mechanism (Fig. 4a, Fig. S4a). Accordingly, immunofluorescence assays were performed to evaluate if Notch2 processing is prevented in Golgi compartment where it undergoes initial enzymatic process. Remarkably, exposure of U-87 MG cells to 2OHOA (400 µM) for 48 h enhanced Notch2 retention in Golgi compartment (marked by Giantin antibody) when the co-localization of these proteins was quantified with the Pearson’s correlation coefficient (Fig. 4b, c). Thus, 2OHOA appears to induce the accumulation of the non-processed Notch2 receptor in Golgi, preventing it from activating this signaling pathway in GBM.

**Fig. 5:**
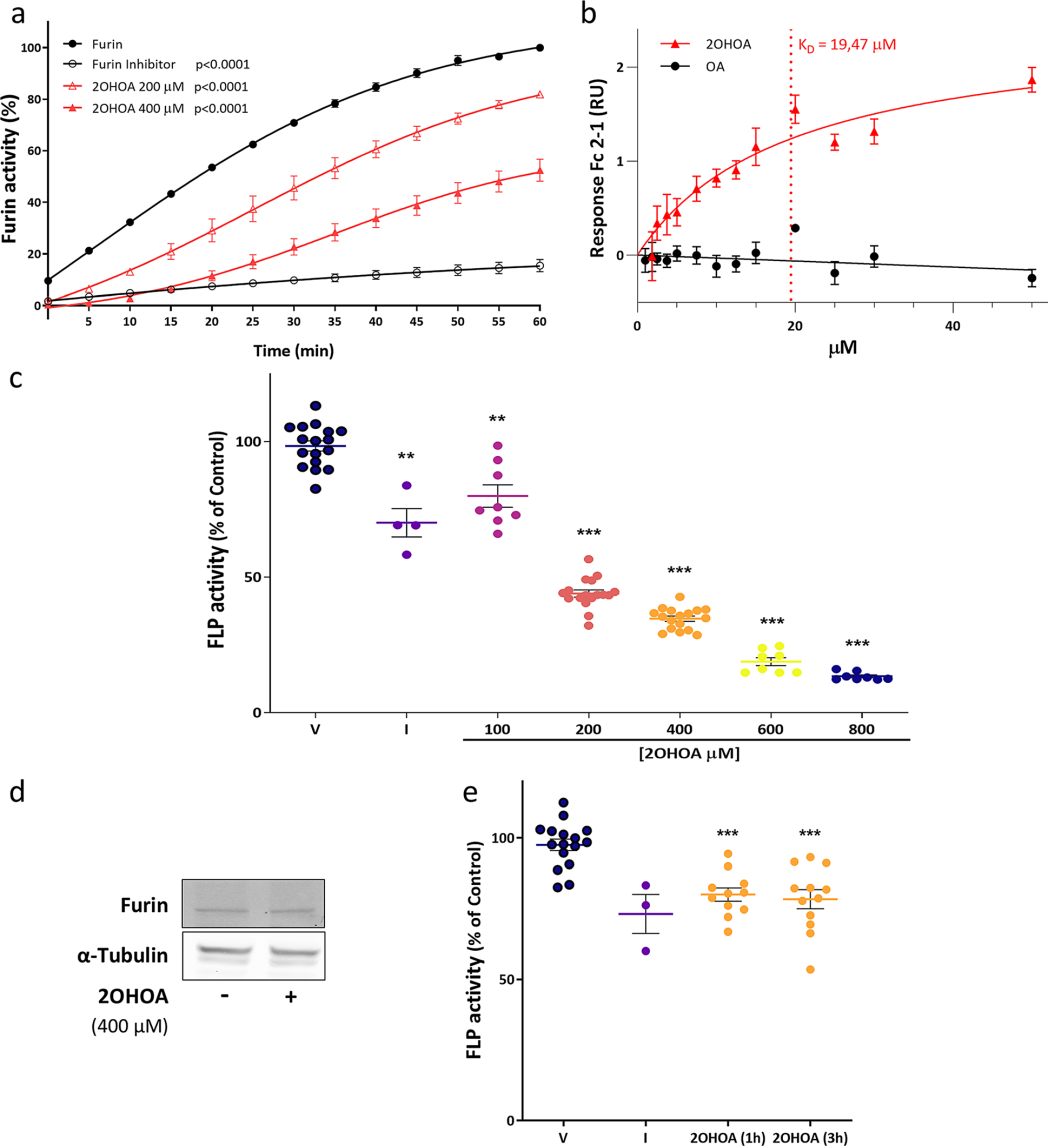
2OHOA inhibits furin activity by physical association. **a** Furin activity assayed in vitro in the presence of 200 and 400 µM 2OHOA, the vehicle alone (ethanol: water, 50:50) or 0.2 µM of the furin inhibitor [peptidyl chloromethylketone (Decanoyl-Arg-Val-Lys-Arg-CMK)]. The results from two independent experiments with three replicates each are expressed as the % of the control ± SEM. The stadistical analysis was determined by a two-tailed Student’s *t* test: ***p < 0.001. **b** SPR measurements of furin interactions with 2OHOA and oleic acid (OA). The SPR response to increasing concentrations of 2OHOA (red circles) and OA (black circles) up to 50 µM. Higher concentrations lead to a response above the theoretical 1:1 Rmax due to lipid aggregation. It was possible to determine a 2OHOA affinity constant at low micromolar levels, yet no interaction of furin with OA was found at the same concentrations. The data points were obtained from three independent experiments run in triplicate, with error bars representing the standard deviation. The dotted line indicates the affinity constant (K_D_=19.47 µM). **c** FLP activity was determined by monitoring boc-RVRR-amc cleavage in U-87 MG cells following exposure to increasing concentrations of 2OHOA (100, 200, 400, 600 and 800 µM) for 48 h, to the vehicle alone (V), or to 100 nM of peptidyl chloromethylketone as a furin inhibitor (I). The values are expressed as the mean ± SEM of three independent experiments analyzed in quadruplicate. **d** Representative immunoblot of furin protein after a 48 h exposure of U-87 MG cell line to 2OHOA (400 µM). **e** FLP activity in U-87 MG cells in response to 2OHOA (400 µM, 1 and 3 h), the vehicle alone or the furin inhibitor (100 nM). The data are expressed as the mean ± SEM of at least two independent experiments analyzed in quadruplicate with a Welch’s *t*-test: **p < 0.01; ***p < 0.001

In light of the above, we consider testing if the impairment of full-length Notch2 processing and trafficking may be due to a furin dysfunction. This protease is responsible for the proteolytic maturation of the Notch receptor upon activation of its signaling pathway in Golgi. Moreover, it has been associated with pathophysiological events. Thus, furin-like protease activity was evaluated in vitro using a fluorimetric kit, following the time course kinetics of rhodamine 110 (Rh110) fluorescence every 5 min for 60 min in the presence of 2OHOA (200 or 400 µM) or its vehicle alone (ethanol: water, 50:50). The loss of fluorescence reflected the inhibition of furin activity in the presence of 200 µM (18.1% ± 0.94) and 400 µM 2OHOA (47.51% ± 4.29) relative to the recombinant human furin mixed with its Rh110 substrate taken as 100% (Fig. 5a).

These results from the in vitro enzymatic reaction not only suggested that 2OHOA dampened the proteolytic activity of furin in a concentration-dependent manner but also, that this might be through a direct interaction. Biacore assays were performed to study this possible protein-lipid interaction, using OA as a control due to its high non-specific affinity of the lipids for the support. Both 2OHOA or OA were used at a concentration up to 30 µM to avoid the formation of micelles [34], and direct interactions of furin with 2OHOA and OA were measured by SPR. It was only possible to obtain a saturation curve in the case of 2OHOA and therefore calculate its affinity, establishing a steady state affinity constant of 2OHOA for furin of 19.47 µM (Fig. 5b). By contrast, it was impossible to obtain any meaningful binding of OA to furin at the tested concentrations, even up to 100 µM (data not shown).

Considering these results, we determined the furin-like protease (FLP) activity in U-87 MG and U-118 MG cells exposed to different concentrations of 2OHOA for 48 h through the conversion of a fluorogenic substrate, boc-RVRR-amc. Enzymatic activity was inhibited significantly in a concentration-dependent manner in U-87 MG but not in U-118 MG cells. Enzyme inhibition (18.47% ± 4.53) was first detected at the lowest tested concentration (100 µM), and it was almost completely inactive following exposure to 800 µM 2OHOA (85.01% ± 1.96: Fig. 5c). Instead, enzyme activity in U-118 MG cells was only inhibited marginally (17.57% ± 2.86) at all 2OHOA concentrations proved (Fig. S4b). This difference could be due to the two-fold greater basal FLP activity in U-87 MG cells than in U-118 MG cells (Fig. S4c). Furthermore, we assessed whether 2OHOA might inhibit furin activity by decreasing the total furin protein in both cell lines, yet no such difference in total furin was detected in these cells in immunoblots when they were treated with 2OHOA (400 µM) for 48 h relative to the controls (Fig. 5d, Fig. S4d). Hence, the inhibition of furin activity in the presence of 2OHOA does not appear to be due to a decrease of the enzyme levels.

FLP activity was also measured 1 and 3 h after of 2OHOA exposure (400 µM) to evaluate if its effects on the Notch2 signaling pathway could be triggered by inhibiting furin activity. Furin activity was clearly hindered by a 1 h exposure of U-87 MG (19.07 ± 3.42) or U-118 MG cells (13.64 ± 3.58) to 2OHOA (Fig. 5e, Fig. S4e), suggesting that the bioactive lipid first acts to inhibit this enzyme that subsequently downregulates Notch2 signaling by preventing its processing, thereby hindering the Notch2 receptor from reaching the membrane.

## Discussion

Patients with GBM have a poor median survival rate due to the high malignancy of these tumors and their resistance to current therapies [4]. In this context, a synthetic derivative of oleic acid, 2OHOA (2-hydroxyoleic acid or 2-hydroxy-9-cis-ocadecenoic acid), was seen to offer efficacy in different animal models as a stand-alone or combination therapy (for instance with TMZ). Due to the hydroxyl group added to its alpha carbon, 2OHOA undergoes degradation by α-oxidation [35, 36] and simultaneously, it inhibits mitochondrial β-oxidation and impairs oxidative phosphorylation, thereby altering the cell’s energetic balance [37]. Significantly, it has successfully completed 2 clinical trials in patients with advanced tumors as a rescue treatment (clinicaltrials.gov Identifier: NCT04250922 [38]; NCT03867123). In all instances, it has displayed a high safety profile both as monotherapy and in conjunction with radio-chemotherapy and has also demonstrated promising clinical activity in patients [38]. Indeed, 2OHOA is currently undergoing human phase IIb/III studies to assess its efficacy for newly diagnosed GBM patients in combination with the SoC (clinicaltrials.gov Identifier: NCT04250922).

The mechanism of action of 2OHOA is based on membrane lipid therapy (melitherapy), which involves the regulation of membrane structure and organization, and the ensuing modulation of intracellular signals, such that it is therapeutic for a variety of pathologies [27, 31, 36]. The changes produced in the membrane by this molecule provoke the translocation of Ras to the cytoplasm, inhibiting the MAPK pathway and in turn dampening PI3K/Akt signaling [24, 39]. Considering the relevance of Notch signaling in GBM and that it is mediated by a transmembrane protein, the compartment where 2OHOA is known to induce biophysical changes [24, 26, 36, 40], we set out to study the effect of this molecule on this pathway and its relevance in its mechanism of action as an antitumor drug against GBM. Several studies have shown that the expression of components of the Notch pathway is altered in different grades of glioma [41–43], making this a potential target to treat this pathology. For instance, overexpression of *NOTCH2* contributes to gliomagenesis, and it serves as a negative prognostic marker preventing apoptosis in GBM cells [10]. Furthermore, 71% of GBMs have aberrant expression of the Notch3 receptor associated with overexpression of its target gene, *HES1* [6]. Elevated *HES1* gene expression could trigger an imbalance between proliferation and differentiation, a critical equilibrium for normal development and homeostasis [44]. As this gene is involved in several physiological and biochemical processes (differentiation, proliferation, cell cycle arrest and survival/apoptosis), its activity as a pro-neural gene repressor would prevent differentiation, favoring proliferation and the initiation of some types of cancer like glioma [33].

Considering the relevance of *HES1* expression in the survival of tumor cells and as the main target of Notch signaling, we determined the effect of 2OHOA on the expression of this gene in U-87 MG and U-118 MG GBM cell lines. The inhibition of *HES1* expression after 2OHOA exposure and its ensuing reduced protein levels suggests that 2OHOA diminished the nuclear accumulation of the Hes1 protein partially through the inhibition of the classic regulatory pathway. Accordingly, its activity as a transcriptional repressor of genes involved in differentiation is hindered, consistent with the induction of differentiation and autophagy by this treatment in glioma cells [24]. Surprisingly, the modulation of *HES1* expression by 2OHOA in GBM cell lines was positively correlated with their sensitivity to chemotherapy, as indicated by their IC_50_. As such, cells that responded to 2OHOA (U- 87 MG, SF-268, U-118 MG, SF-295 and U-251 MG) had a higher reduction of the *HES1* mRNA levels upon 2OHOA treatment in comparison with other glioma cell lines (SNB-19 MG, SNB-75 MG) with resistance to the bioactive molecule, indicating the relevance of this gene in the effect of 2OHOA. However, no association was obtained between the IC_50_ and the baseline *HES1* gene expression, suggesting that the mechanism by which 2OHOA inhibits cell proliferation does not depend on the initial *HES1* gene expression but rather, that the antitumor effect may be triggered by the repression of *HES1* expression. Hence, the inhibition of *HES1* gene could be involved in the mechanism of action of 2OHOA. Furthermore, there is evidence of a link between Hes1 and drug resistance, maintaining cells in a quiescent state by preventing senescence through different mechanisms and involving numerous signaling pathways [33, 45]. In this context, *HES1* overexpression partially inhibits the effect of 2OHOA on U-87 MG and U-118 MG cells viability, suggesting that reduced *HES1* expression and its absence as a nuclear transcription factor, as shown in U-87 MG cells, are relevant to the effect of 2OHOA on cell death. As well as the decrease of nuclear Notch3, implicating both proteins in the cell survival and anti-proliferative effects of 2OHOA.

Proof-of-concept studies demonstrated that 2OHOA has a greater antitumor activity than TMZ, with no toxicity at therapeutic doses and no recurrence [24, 27]. The volume of tumors derived from U-118 MG cells in immunosuppressed mice was reduced by 32.07% relative to those treated with the vehicle alone, confirming the efficacy of this molecule in vivo. In the tumors treated with 2OHOA that reduced in size there was a strong positive correlation between *HES1* expression and tumor volume. This correlation was not evident in tumors treated with the vehicle alone, confirming that *HES1* might be a predictive biomarker of efficacy and plays a relevant role in the mechanism of 2OHOA to induce tumor shrinkage in those that respond to this treatment.

Apart from *HES1*, elevated expression of several other members of the Notch pathway has also been correlated with a more aggressive phenotype and a worse prognosis, including *NOTCH1*, *NOTCH2*, *NOTCH3*, *NOTCH4*, *ASCL1* and *HEY* [2, 10, 46]. 2OHOA inhibits Notch signaling by downregulating the transcription of Notch pathway components (*JAGGED1*, *NOTCH1*, *NOTCH3*) and preventing the processing of immature Notch2 receptor in both cell lines, triggering a blockage of Notch signaling and, thus, repressing the expression of the Notch target genes *HES1* and *CD3*. Moreover, it should be noted that CD3 was previously described as a target of 2OHOA due to inhibition of the Ras/MAPK pathway [24], suggesting that this bioactive lipid can downregulate different pathways related to glioma proliferation as part of its anti-tumoral effect. Considering that MAPK pathway downregulates *NOTCH3* expression [47, 48], its transcriptional repression in response to 2OHOA might be induced via inhibition of this pathway. Although overexpression of Notch3 has been reported in 71% of primary GBM associated with aberrant *HES1* expression [6, 46], our data indicates that Notch2 signaling is the predominant pathway in these GBM cell lines, as supported by The Human Protein Atlas database (proteinatlas.org/NOTCH2 for *NOTCH2* and proteinatlas.org/NOTCH3 for *NOTCH3*). Interestingly, the lowest Hes1 protein levels were achieved after inhibiting both homologous proteins expression by using siRNA against *NOTCH2*. However, the downregulation of *NOTCH3* expression with its specific siRNA increased the expression of *NOTCH2* instead of inhibiting it. This was translated as a lower effect on Hes1 protein levels, compared to the transfection with siRNA against *NOTCH2*, highlighting the relevance of both isoforms activity. These data support that 2OHOA is a potent Notch inhibitor due to its capacity to inhibit the activity of both homologous proteins, with a potential therapeutic effect in tumors highly dependent of Notch pathways activity as could be GBM.

As indicated in the introduction, the first cleavage of the Notch receptor (S1) is carried out by a furin protease in the trans-Golgi network, which is responsible for the maturation of full-length Notch into a non-covalent heterodimeric receptor with an extracellular domain linked to the transmembrane/intracellular fragment [14]. This maturation permits receptor translocation to the cell membrane where it can initiate signaling. Since immunofluorescence assays indicated that Notch2 processing was impaired in Golgi, we focused on how 2OHOA affects enzymatic regulation in the processing of this protein. Numerous compounds have been used to inhibit aberrant Notch signaling in cancer by downregulating the enzymes involved in Notch processing. Furin inhibitors are not usual studied, the most employed being those that inhibit the γ-secretase enzyme (GSIs) which processes the final cleavage of the receptor, preventing its translocation into the nucleus and the ensuing transcription of its target genes [2]. DAPT is the most widely used GSI, the anti-proliferative and pro-apoptotic activity of which induces astrocyte and neuronal differentiation [49]. However, cancer cells may be resistant to GSIs by expressing efflux pumps that expel these drugs from the cell. By contrast, α-secretase inhibitors (ASIs) prevent the second cleavage (S2) of the Notch receptor by inhibiting the enzyme that performs this function [A disintegrin and metalloprotease (ADAM) family proteins]. These inhibitors are not affected by such pumps as they act outside of the plasma membrane domain, making them more effective [50]. Notwithstanding, only one ASI is currently running a phase I to treat pediatric high-grade gliomas [51] likely as a result of multiple failures obtained, due to adverse side effects, using ADAM inhibitors in clinical trials [52]. In recent years, furin has become of significant interest promoting human diseases, including pathogenic infections and cancer, when its proteolytic activity is dysregulated [53, 54]. Since Notch precursors require processing by furin before binding with their ligands to activate the signaling pathway, being ADAM members also catalyzed by this enzyme [55] and the insignificant toxicity obtained with 2OHOA in comparison with GSIs and ASIs, we propose furin as a promising target to hindering the Notch pathway.

**Fig. 6:**
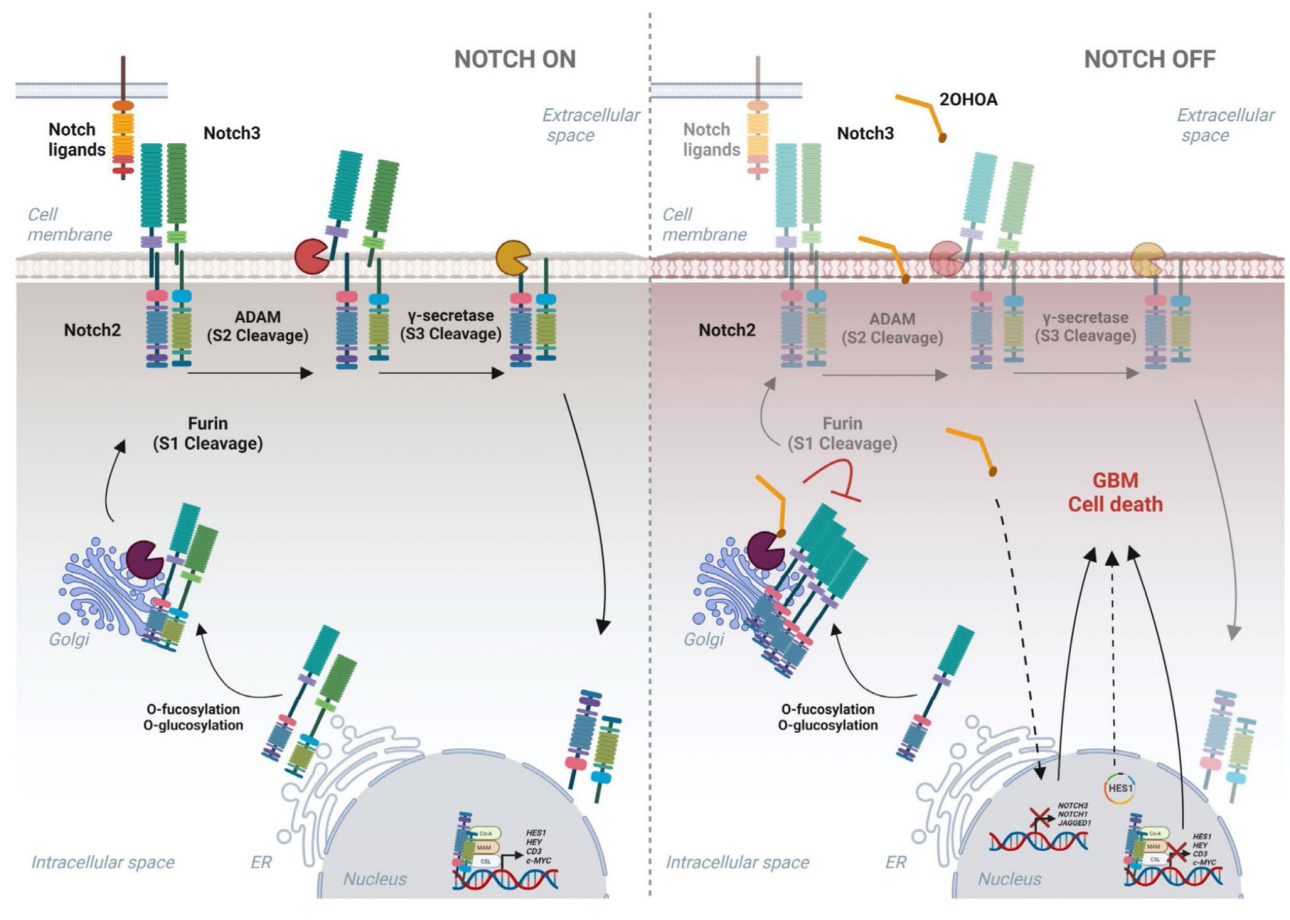
Graphical illustration describing the 2OHOA mechanism of action to inhibit Notch2 and Notch3 signaling. Model for activated Notch2/3 signaling (left panel). Proposal model for 2OHOA mechanism of action (right panel) where its interaction with the furin enzyme prevents Notch2 processing in Golgi. Instead, Notch3 is transcriptionally repressed probably by indirect 2OHOA mechanism (indicated as dashed line). In addition, HES1 overexpression partially inhibits the 2OHOA effect on viability (narrower dashed line). Continuous lines indicate activation while transparent continuous lines represent lower activation of the pathway

Regarding our findings, furin-like protease activity was evaluated in vitro and seen to be inhibited in a concentration-dependent manner. In vivo, 2OHOA downregulates furin activity in GBM cell lines after 1 h of treatment, with a stronger concentration-dependent effect on U-87 MG cells after 48 h treatment, while the effect was independent of dose in U-118 MG cells. These differences may be reflected by different basal states of furin activation, showing a two-fold greater protease activity in U-87 MG cells than in U-118 MG cells. Biacore binding detected a direct interaction between 2OHOA and furin, indicating this to be the mechanism by which this protease activity is inhibited as a consequence of the addition of a hydroxyl group to the alpha carbon of 2OHOA, identifying it as a novel target for this drug. The ensuing integration appears to induce the accumulation of immature Notch2 receptor in Golgi, preventing it from activating this signaling pathway in GBM (Fig. 6). All together reveals that the inhibition of Notch pathway by 2OHOA plays a role in its antitumoral effect, this event being unleashed by the direct furin enzyme inhibition and by the repression of genes related to activate Notch pathway in GBM.

## Conclusions

2OHOA is a promising therapeutic compound to treat GBM in vitro and in vivo, as witnessed in cultured cancer cell, animal models, and patients recruited in different clinical trials (phase I/IIa, phase Ib and running phase II/III). As it has been described, its mechanism of action is based on melitherapy altering the activity of membrane-associated proteins such as Notch. Our findings demonstrate the inhibition of Notch2 and Notch3 signaling by 2OHOA in GBM cell lines was induced through dual mechanism. Notch3 signaling is abolished by repressing its transcription. Instead, Notch2 pathway is hindered by blocking its first cleavage. The mechanism of action is induced by inhibition of furin activity through a direct physical interaction, characterizing a novel target for this bioactive molecule. Therefore, Notch2 processing is impaired, and the non-functional receptor is retained in the Golgi complex, preventing its trafficking to the plasma membrane to initiate the signaling cascade. Consequently, Notch2 and Notch3 signaling is downregulated and genes whose transcription are NICD2/3 dependent are repressed. Finally, the relevance of this pathway in the 2OHOA pharmacological efficacy was emphasized not just through the overexpression of its principal target, *HES1*, which partially dampened the drug’s effect on viability. Additionally, the drug-induced reduction in *HES1* expression exhibited a positive correlation with increased sensitivity to the molecule. Collectively, it becomes evident that the antitumoral effect of 2OHOA involves the inhibition of the Notch pathway as a part of the 2OHOA mechanism of action against GBM.

## Electronic supplementary material

Below is the link to the electronic supplementary material.

Supplementary Material 1

## Data Availability

All the data generated or analyzed in this study are included in the manuscript and Supplementary Information except for binding affinities of furin to OA in concentrations above 50 µM, which are available from the corresponding author upon request. The data that support NOTCH2 expression is higher than NOTCH3 in the different brain cancer cell lines are openly available in The Human Protein Atlas website (proteinatlas.org/NOTCH2 for NOTCH2 and proteinatlas.org/NOTCH3 for NOTCH3).

## Abbreviations

2OHOA: 2-hydroxyoleic acid/2-hydroxy-9-cis-ocadecenoic acid
ADAM: A disintegrin and metalloprotease domain
ASI: α-secretase inhibitor
DAPI: 4′,6-Diamidino-2-phenylindole
DAPT: (N-[N-(3,5-difluorophenacetyl)-l-alanyl]-s-phenylglycinet-butyl ester)
FBS: Fetal Bovine Serum
FLP: Furin-like protease
GBM: Glioblastoma multiforme
GSI: γ-secretase inhibitor
HES1: Hairy and enhancer of split-1
I: Inhibitor
IC_50_: Half maximal inhibitory concentration
JACoP: Just another co-localization plugin
H: Hour
IR: Insulin receptor
μM: Micromolar
Min: Minute
Melitherapy: Membrane lipid therapy
Notch2/3 FL: Notch2/3 Full-length
Notch2/3 TM: Notch2/3 Transmembrane
NICD2/3: Notch2/3 intracellular domain
mRNA: Messenger RNA
OA: Oleic acid
PB: Phosphate buffer
PBS: Phosphate-buffered saline
PFA: Paraformaldehyde
qRT-PCR: Real‑Time Quantitative PCR
RFU: Relative fluorescence unit
Rh110: Rhodamine 110
S1: First cleavage
S2: Second cleavage
siRNA: Small interfering RNA
SoC: Standard of care
SPR: Surface plasmon resonance
TMZ: Temozolomide
T-TBS: Tween-Tris-buffered saline
V: Vehicle
