# Supplementary material for: Targeting the Notch-Furin axis with 2-hydroxyoleic acid: a key mechanism in glioblastoma therapy
Source: Cell Oncol (Dordr). 2024 Oct 14;48(2):373–90. doi: 10.1007/s13402-024-00995-x (PMC11996967; doi:10.1007/s13402-024-00995-x)
Supplement: Supplementary file 1 — Supplementary Material 1 [file 13402_2024_995_MOESM1_ESM.docx]

**
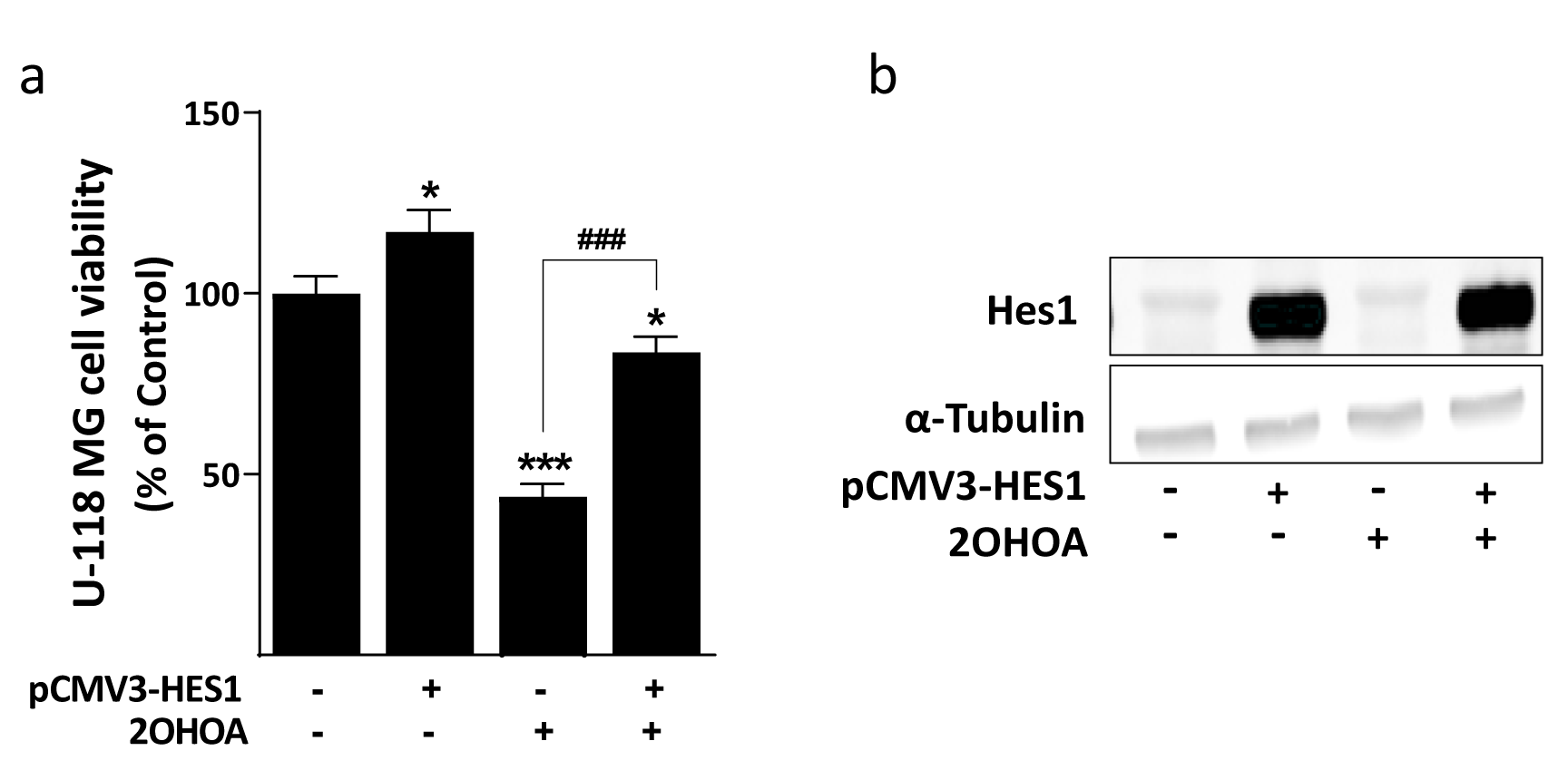
**

**Fig. S1.** *HES1* expression is also involved in the mechanism of action of 2OHOA in U-118 MG cell line. **(a)** Viability of U-118 MG cells transfected with 1 µg of empty plasmid (control) or pCMV3-HES1 for 24 hours prior to 2OHOA treatment (200 μM for 48 hours) as determined by trypan blue exclusion. The data are expressed as the % of the control ± S.E.M of three independent experiments with three replicates each. The statistical analysis was performed with a t-test with Welch’s correction relative to cells transfected with the empty plasmid: *p < 0.05, ***p < 0.001 or pCMV3-HES1: ^###^p < 0.001. **(b)** Transfection efficiency of pCMV3-HES1 into U118-MG cells as seen in Western Blots.


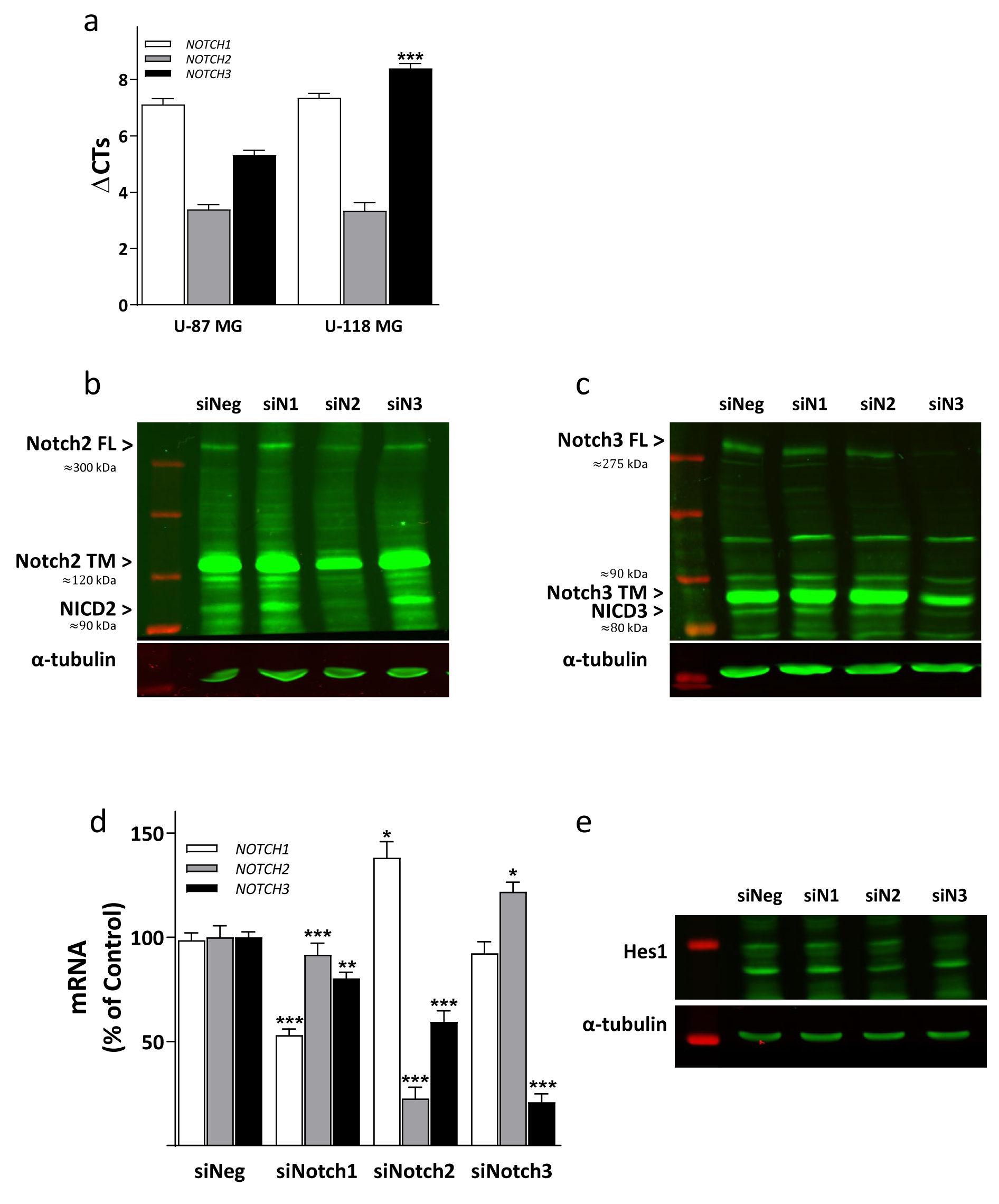


**Fig. S2.** Characterization of Notch2 and Notch3 homolog proteins. **(a)** Quantitative Real-Time PCR (qRT-PCR) data showing Delta Cts for *NOTCH1*, *NOTCH2* and *NOTCH3* genes in U-87 MG and U-118 MG. Delta Cts were obtained from three independent experiments analyzed in triplicate using the formula ∆Ct = Ct (gene of interest) – Ct (housekeeping gene). The statistical analysis was performed per each gene between cells with a t-test with Welch’s correction: ***p < 0.001. **(b, c)** Western blot analysis of Notch2 (b) and Notch3 (c) antibodies in transfected U-87 MG cells with 20 nM of siNotch1 (siN1), siNotch2 (siN2), siNotch3 (siN3) or non-specific siRNA (siNeg) for 72 h. **(d)** The expression levels of *NOTCH1*, *NOTCH2* and *NOTCH3* mRNA to assess the efficiency of cell transfection. The values obtained by qRT-PCR from three independent experiments with at least two replicates each. The statistical analysis was performed with a t-test with Welch’s correction relative to non-specific siRNA transfected cells: *p < 0.05, **p < 0.01, ***p < 0.001. **(e)** Relevance of Notch2 by western blot analysis of Hes1 under the same transfection conditions as in b, c.


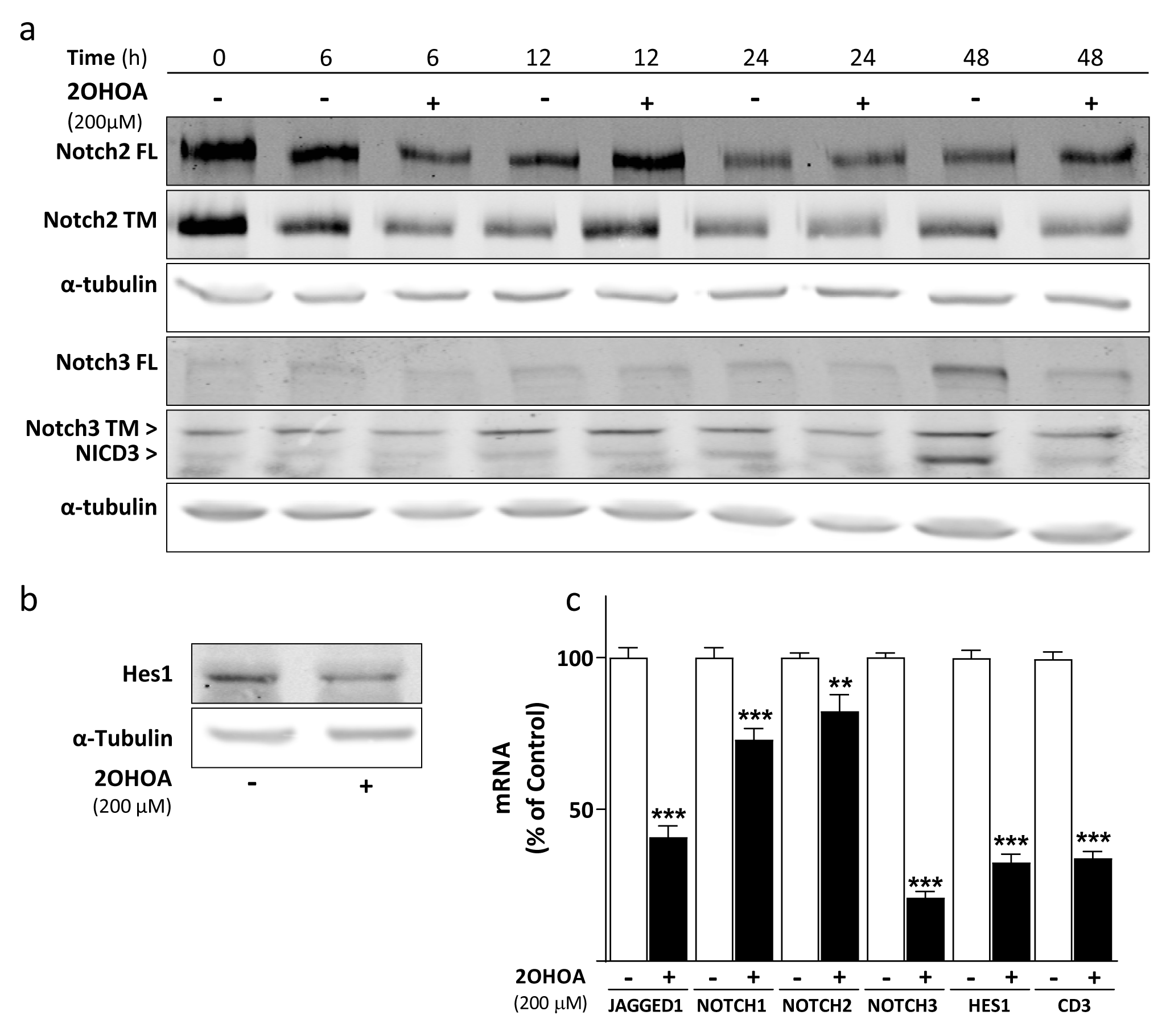


**Fig. S3.** 2OHOA inhibits Notch signaling pathway as well in U-118 MG. **(a)** Representative immunoblot of Notch2/3 FL (full length), Notch2/3 TM (transmembrane domain) and NICD3 (Notch intracellular domain) protein after exposure of U-118 MG cells to 2OHOA (200 µM) for 6, 12, 24 and 48 hours (four independent experiments with two replicates each). **(b)** The Hes1 protein levels after exposure to 2OHOA (200 µM) for 48 h as an indicator of Notch pathway inhibition in U-118 MG cells. **(c)** The mRNA expression of *JAGGED1*, *NOTCH1*, *NOTCH2*, *NOTCH3*, *HES1* and *CD3* genes in U-87 MG treated for 48 h with 2OHOA (200 µM) or the vehicle alone. The values are presented as the mean ± SEM of at least four independent experiments analyzed in triplicate. The statistical analysis was performed with a t-test with Welch’s correction relative to the untreated cells: ***p < 0.001.


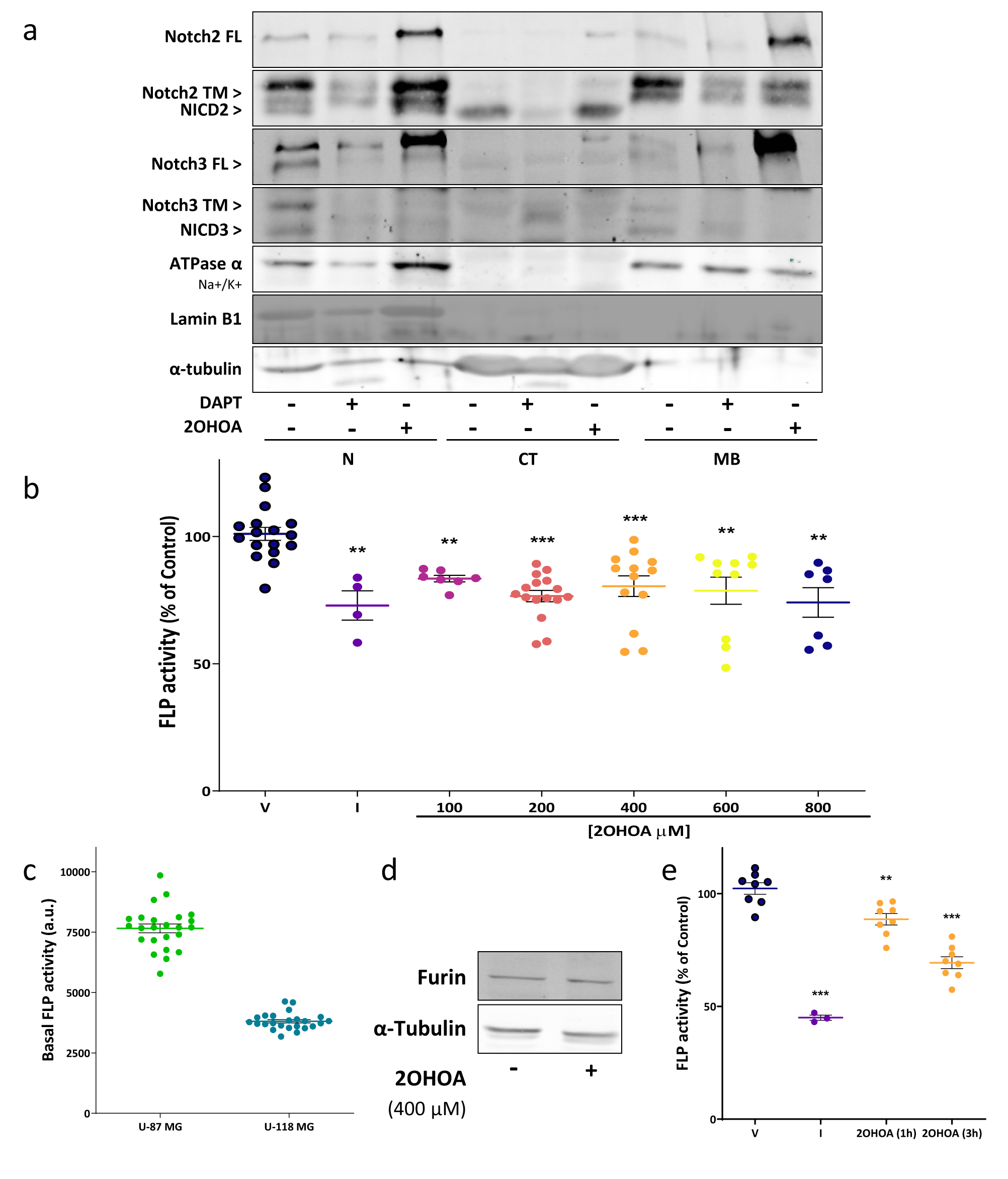


**Fig. S4.** 2OHOA prevents Notch2 processing and trafficking by direct furin activity inhibition. **(a)** Representative immunoblot of the U-118 MG subcellular fractionation from six independent experiments with two replicates each. The distribution of the Notch protein: Notch2/3 FL, Notch2/3 TM and NICD2/3 in cells maintained for 48 h in the presence or absence of 2OHOA (400 μM) or DAPT (10 μM, 24 h), a positive control for the loss of NICD, is shown in the different cellular compartments: N, nucleus; CT, cytosol; and MB, total membranes. The efficiency of fractionation was assessed by analyzing reference proteins from the different compartments: Laminin B1 (nucleus), tubulin (cytosol) and ATPase α (Na+/K+) for the membranes. **(b)** FLP activity was determined by monitoring boc-RVRR-amc cleavage in U-118 MG cells following exposure to increasing concentrations of 2OHOA (100, 200, 400, 600 and 800 µM) for 48 h, to the vehicle alone (V), or to 100 nM of peptidyl chloromethylketone as a furin inhibitor (I). The values are expressed as the mean ± SEM of three independent experiments analyzed in quadruplicate. **(c)** Basal activity of FLP in U-87 MG and U-118 MG. **(d)** Immunoblot of furin protein after a 48 h exposure of U-118 MG to 2OHOA (400 µM). **(e)** FLP activity in U-118 MG in response to 2OHOA (400 µM, 1 and 3 h), V or I. The data are expressed as the mean ± SEM of at least two independent experiments analyzed in quadruplicate. The statistical analysis was performed with a t-test with Welch’s correction relative to the untreated cells: ***p < 0.001.

**Table S1.** *HES1* expression in response to 2OHOA correlates with its pharmacological potency. **(a)** Correlation analysis between the IC_50_ values and *HES1* basal expression (column 2) or in response to 2OHOA (column 3). Statistical significance was determined with a Student's t-test: *p<0.05. **(b)** *HES1* mRNA values at baseline (column 3) or in response to a 24 h exposure to 2OHOA (200 µM, column 4) of different glioma cell lines (column 1) with the respective IC_50_ values (column 2): mRNA expression was assessed by qRT-PCR from 2 experiments with 4 replicates each. The treatment response is expressed as a percentage of the control ± SEM, while the baseline values are expressed in arbitrary units ± SEM after quantification using a standard curve.

**a**

| **Pearson r** | **IC_50_ (μM) vs. *HES1* mRNA Basal expression** | **IC_50_ (μM) vs. *HES1* mRNA in response to 2OHOA** |
| --- | --- | --- |
| r | 0.4066 | 0.8211 |
| 95% confidence interval | -0.4994 to 0.8878 | 0.1782 to 0.9727 |
| R^2^ | 0.1653 | 0.6742 |
| P value (one-tailed) | 0.1827 | 0.0236 |
| P value summary | ns | * |
| α= 0,05 | No | Yes |
| N | 7 | 7 |

**b**

| **Cell line** | **IC_50_ (μM)** | ***HES1* mRNA Basal expression (a.u)** | ***HES1* mRNA in response to 2OHOA (%)** |
| --- | --- | --- | --- |
| SNB-19 | 800.00 | 7.71 ± 0.55 | 138.30 ± 18.85 |
| SNB-75 | 800.00 | 1.00 ± 0.24 | 99.59 ± 8.51 |
| U-87 MG | 171.90 | 0.35 ± 0.01 | 74.32 ± 3.01 |
| SF-295 | 300.00 | 2.34 ± 0.27 | 57.58 ± 3.31 |
| SF-268 | 240.00 | 0.40 ± 0.04 | 52.77 ± 9.59 |
| U-251 MG | 426.51 | 6.19 ± 0.09 | 45.55 ± 3.90 |
| U-118 MG | 180.1 | 3.93 ± 1.69 | 39.47 ± 4.19 |

**Table S2.** *HES1* expression in response to 2OHOA also correlates with its pharmacological potency *in vivo*. *HES1* expression relative to tumor volume and treatment. Each value represents the mean ± SEM of 8 mice per group analyzed in duplicate by qRT-PCR.

| **Group** | **Tumor volume (%)** | ***HES1* expression (a.u.)** |
| --- | --- | --- |
| Vehicle | 420.59 | 1.49 ± 0.09 |
|  | 442.17 | 1.15 ± 0.12 |
|  | 424.88 | 1.13 ± 0.02 |
|  | 619.31 | 1.10 ± 0.07 |
|  | 384.66 | 1.03 ± 0.1 |
|  | 832.46 | 0.97 ± 0.03 |
|  | 1212.55 | 0.70 ± 0.09 |
|  | 878.83 | 0.69 ± 0.04 |
| 2OHOA | 333.98 | 1.32 ± 0.2 |
|  | 359.43 | 1.28 ± 0 02 |
|  | 280.83 | 1.27 ± 0.07 |
|  | 1251.02 | 1.09 ±0.09 |
|  | 509.20 | 1.02 ± 0.12 |
|  | 103.36 | 0.99 ± 0.05 |
|  | 100 | 0.75 ± 0.07 |
|  | 67.82 | 0.53 ± 0.06 |
